# Attachment Styles and Suicide-Related Behaviors in Adolescence: The Mediating Role of Self-Criticism and Dependency

**DOI:** 10.3389/fpsyt.2017.00036

**Published:** 2017-03-10

**Authors:** Giorgio Falgares, Daniela Marchetti, Sandro De Santis, Danilo Carrozzino, Daniel C. Kopala-Sibley, Mario Fulcheri, Maria Cristina Verrocchio

**Affiliations:** ^1^Department of Psychological, Pedagogical and Educational Sciences, University of Palermo, Palermo, Italy; ^2^Department of Psychological, Health and Territorial Sciences, University of Chieti-Pescara, Chieti, Italy; ^3^Azienda Provinciale per i Servizi Sanitari, Provincia Autonoma di Trento, Trento, Italy; ^4^Department of Psychology, Stony Brook University, New York, NY, USA

**Keywords:** attachment, depressive experiences questionnaire for adolescents, personality, suicide, mediation effect

## Abstract

Insecure attachment and the personality dimensions of self-criticism and dependency have been proposed as risk factors for suicide in adolescents. The present study examines whether self-criticism and dependency mediate the relationship between insecure attachment styles and suicidality. A sample of 340 high-school students (73.2% females), ranging in age from 13 to 20 years (M = 16.47, SD = 1.52), completed the Depressive Experiences Questionnaire for Adolescents, the Depressive Experiences Questionnaire for Adolescents, the Attachment Style Questionnaire, and the Suicidal Behaviors Questionnaire-Revised. The results partially support the expected mediation effects. Self-criticism, but not dependency, mediates the link between insecure attachment (anxiety and avoidance) and suicide-related behaviors. Implications for suicide risk assessment and management are discussed.

## Introduction

Suicidal ideation and behavior among adolescents is an emerging global public health problem (1). According to the Italian National Institute of Statistics, Italy is among the European countries with the lowest levels of mortality by suicide, despite suicide being the second leading cause of death among men aged 15–29 years and the third leading cause of death among women of the same age range (2).

Among potential risk factors implicated for suicidal ideation (i.e., thoughts about engaging in behaviors that are intended to end one’s life) and attempts (i.e., deliberately causing harm to oneself with at least some intent to die) in adolescents (3, 4), empirical research has focused on a range of factors, including drug and alcohol abuse, caregiver suicide attempts (5), previous suicide attempts, and experiences of childhood abuse and neglect (6). Other studies have examined school problems, such as bullying or academic failure (7, 8), as well as interpersonal (9) and familial difficulties including frequent criticism, poor communication, perceived lack of support (10, 11), peer rejection, and low social support from friends (12). Still other studies have examined intrapersonal markers of risk, including impulsivity, rumination, hopelessness, mental pain (13–16), hostility (17–19), and the presence of a chronic disease, particularly depression (20).

Moreover, theory and research both suggest that two important personality vulnerability factors, namely dependency and self-criticism [e.g., Ref. (21–23)] and early developmental vulnerabilities, in particular, insecure attachment [e.g., Ref. (10, 23, 24)] confer vulnerability for suicidality in adolescents. However, to our knowledge, the literature to date pertaining to dependency and self-criticism has not evaluated them as clinical factors linking the potential association between attachment and suicidality in adolescents. Thus, the present study aims to evaluate the association between attachment styles, personality vulnerability dimensions, and suicide-related behaviors during adolescence.

Specifically, in order to examine the relationship between attachment and suicide risk, we focused on the possible mediating effect of the personality traits of self-criticism and dependency according to Blatt’s two-configurations model (21, 25).

### The Associations between Blatt’s Two-Configurations Model and Suicidality

According to Blatt’s two-configurations model (26, 27), personality proceeds through a dialectical and continuing interaction between the issues of identity, autonomy, and achievement on the one hand, and interpersonal issues of relatedness, attachment, and intimacy, on the other (28). It has been proposed that this model may contribute to our understanding of vulnerability to suicide in adolescents and adults as it may elucidate specific patterns of risk (22, 29, 30), further, our knowledge of the etiology of suicidal behaviors, and improve our treatments for suicidal patients (31).

Mature personality can be considered a synergistic product of these two developmental configurations that extend throughout life: interpersonal relatedness, which involves developing the capacity for mature, intimate, reciprocal, and mutually satisfactory interpersonal relationships, and self-definition, which involves the development of a realistic, integrated, and differentiated identity or sense of self (32). Even in normal development, individuals usually place an emphasis on one dimension, creating two basic personality styles. For some, the emphasis is on interpersonal relatedness and is more focused on the desire for harmonious, supportive, and reciprocal relationships. For others, there is an emphasis on self-definition, which is more focused on individuation, achievements, and identity formation (21).

A disruption in this normal developmental dialectic process may result in a rigid, one-sided preoccupation with one of these two dimensions at the expense of the other. In particular, an overemphasis on issues of relatedness is the basis of a pathological personality style that Blatt labeled *dependent/anaclitic*. An overemphasis on issues of self-definition is the basis of a pathological personality style labeled *self-criticism* (33).

According to Blatt (34), high levels of dependency and/or self-criticism can confer vulnerability to two different forms of depression. Specifically, dependent individuals are characterized by preoccupations with issues of closeness, affiliation, and interpersonal connectedness; these individuals are especially sensitive to situations of separation and loss and tend to respond to such situations with feelings of helplessness and emptiness. In contrast, self-critical individuals are particularly concerned about experiences of shame and personal failure. Highly self-critical individuals tend to experience feelings of guilt and self-blame during instances of perceived failure and are particularly prone to depression in these contexts (35).

Research has systematically demonstrated that the pathological personality traits of dependency and self-criticism are related to depression, which is in turn linked with suicidality (22). It has also been proposed that dependent and self-critical individuals may display different types of suicidal behaviors, similar to the differences shown by these two personality types with regard to depression (36). Fehon et al. (13), for example, examined associations between dependency, self-criticism, impulsivity, and suicidal behavior in a sample of adolescent patients. Although they (13) found that suicide risk did not greatly differ between highly self-critical and highly dependent patients, dependent individuals appeared generally to engage in patterns of impulsive gestures and attempts, whereas self-critical individuals appeared less impulsive and more likely to plan acts of self-harm.

Fazaa and Page (36) found that more highly self-critical patients were more likely to have made a suicide attempt in response to a personal or professional failure, and that their intention in attempting suicide was to escape from the actual events–expectations discrepancy (37), and that their suicide attempts on the whole were rated as more lethal than more relatively dependent patients. Dependent suicide attempters were more likely to have made their attempt in response to an interpersonal stressor and indicated that their intention in attempting suicide was to communicate their feelings of distress to others.

Fazaa and Page (38), also, found that adult participants with higher levels of dependency showed higher rescue scores (i.e., using methods that made rescue more likely), compared to those scoring lower on dependency. In contrast, higher levels of self-criticism were associated with increased suicidal intent (i.e., greater wish to die), compared to those scoring lower on self-criticism. In an adult sample, Campos et al. (22) found that depressive symptoms mediated the association between self-critical perfectionism and suicidality. Highly self-critically perfectionistic individuals are vulnerable to intense depression, often accompanied by suicidal impulses, when confronted with stressful life events, and, in particular, events that disrupt self-definition and/or a sense of personal achievement.

Campos and Mesquita (20) tested a model of suicidality that included dependency, self-criticism, anger-temperament, depression, and anger-in (i.e., the expression of anger against the self) in a community of adolescents. Self-critical, dependent, and anger-in traits predicted depression, which in turn predicted suicidality directly and indirectly through anger-in. Similarly, Campos and Holden (39) showed that, even within a sample of depressed adults, elevated self-criticism is associated with a greater likelihood of suicidal behaviors.

Finally, Campos et al. (40) found that depression and social withdrawal mediated the relationship between both dependent and self-critical vulnerabilities and suicidality in a community sample of adolescents.

In summary, much theory and evidence in adolescents has linked the personality traits of self-criticism and dependency to suicidal ideations and behaviors, which suggests that they may be important etiological components of risk for suicide.

### Attachment Dimensions As Risk Factors for Suicide among Adolescents

Maimon et al. (41) identified family attachment as a protective factor against adolescent suicide attempts. Similarly, evidence consistently indicates that disrupted parent–child interactions play a significant role in the development of a self-critical or dependent vulnerability to depression [see Ref. (32), for a review], which is in turn an important factor for suicide risk for adolescents (22, 42, 43).

Attachment theory argues that early experiences with caregivers are translated into internal working models that guide individuals’ understanding of relationships across the lifespan (44, 45). Insecure attachment is relevant to adolescent suicidal behavior because it is associated with relationship dysfunction (46), which often precedes adolescent suicide attempts (47).

Indeed, Violato and Arato (48) showed that preoccupied and disorganized attachment was associated with suicidal behavior among adolescents in psychiatric treatment. Among undergraduates, a history of suicide ideation or attempts was associated with low attachment security, whereas preoccupied and dismissing attachments predicted suicidality.

Based on findings of Sheftall et al. (49), suicide attempters reported significantly higher greater attachment avoidance and anxiety. Attachment avoidance, but not anxiety, predicted suicide attempt status in a conditional logistic regression analysis that controlled for depressive symptoms and family alliance.

In contrast, however, Venta and Sharp (10) found no relation between attachment organization and suicidal thoughts and behavior. Instead, they confirmed the relation between internalizing disorders and a lifetime history of self-harm, suicide ideation during the past year, and lifetime suicide attempts, whereas externalizing disorders were associated with increased lifetime self-harm. They suggest that the link between attachment organization and suicidal thoughts and behavior may be mediated by other factors.

### The Role of Self-Criticism and Dependency in the Link between Attachment Styles and Suicidality

Despite evidence that self-criticism, dependency, and attachment dimensions are distinguishable constructs (43), Blatt’s two-configurations model and attachment theory both posit that personality functioning involves a balance between relatedness and self-definition expressed in low to moderate levels of attachment anxiety and avoidance typical of secure attachment (50). Maladaptive personality functioning, in contrast, typical of insecure attachment, results from an overemphasis of relatedness/attachment anxiety or self-definition/attachment avoidance or both (21).

Specifically, the attachment avoidance dimension, defined in terms of “discomfort with closeness and with discomfort depending on others” [(51), p. 87], is conceptually related to the self-definition dimension. Attachment anxiety, in contrast, defined in terms of “fear of rejection and abandonment” [(51), p. 91], is conceptually related to the relatedness dimension. Many studies have empirically confirmed these hypotheses. For example, Zuroff and Fitzpatrick (52) found an association between self-criticism and fearful-avoidant styles and between dependency and anxious attachment styles. Specifically, results in Zuroff and Fitzpatrick (52) as well as other showed that attachment anxiety was positively correlated with dependency while avoidance was positively related with self-criticism (51–55). Major features of attachment anxiety include the desire for interpersonal closeness and a fear of interpersonal rejection or abandonment (56). Therefore, these individuals may develop a dependent tendency in order to ensure others’ availability and validation.

Conversely, research suggests that those with higher levels of attachment avoidance may be able to prevent psychopathological symptoms by avoiding dependence (52, 54). This also supports the theoretical perspective that those with higher levels of attachment avoidance have learned that others are untrustworthy. As a result, they have learned to rely on themselves instead of others in order to prevent hurt or disappointment. In sum, avoidantly attached individuals may actively avoid being dependent on others and, instead, strive for autonomy and independence, two values that are important to highly self-critical people. Moreover, avoidantly attached people have a negative working model of themselves or a poor sense of self-worth and often have a negative working model of others (57), which is similar to how self-critical individuals are often critical of both themselves and others (58).

To our knowledge, no studies have specifically investigated the mediating role of Blattian variables among attachment styles and suicidality, although some have focused on the influence of parenting on suicidality *via* self-criticism and dependency.

For example, Quinlan et al. (59) found that individual descriptions of both parents as less benevolent and more punitive correlated positively with self-criticism. The findings indicate that perceived dysfunctional early relationships with caregivers is associated with self-criticism as well as with depression and suicidal behavior.

Moreover, Campos et al. (22) examined whether self-criticism and depressive symptoms mediate the relationship between recollections of parental rejection and suicidality. Findings indicate that recollections of parental rejection are significantly associated with suicidality and depressive symptoms and that recollections of parental rejection are also indirectly associated with suicidality and depression through self-criticism. Moreover, the association between self-criticism and suicidality was mediated by depressive symptoms.

In sum, we expect the Blattian traits of self-criticism and dependency to mediate already well-known relationship between attachment and suicidality. Specifically, we expect self-criticism to mediate the link between both attachment anxiety and avoidance, and suicidality, whereas we expect dependency to mediate the link between attachment anxiety and suicidality. This study stands to provide relevant clinical information regarding the distinct motivations (particularly in terms of different vulnerability factors) that render individuals prone to suicidal crises.

## Materials and Methods

### Participants

Four hundred three high school students from three schools in Palermo (Italy) were invited to participate: 41 did not accept and thus, 362 participated. Of these, 10 (2.76%) were eliminated because the questionnaires were not completed. Specifically, missing data for one or more variables were replaced with the mean of the scale where they did not exceed 20%. If in every questionnaire, missing data exceeded this cutoff, they were excluded from the analyses. The questionnaires were also excluded if there were univariate outliers (*N* = 12; *z* scores >3). There were no multivariate outliers (scores did not exceed the Kurtosis multivariate Mardia coefficient, equal to 80).

The final sample consisted of 340 participants (73.2% female; M_age_ = 16.47, SD = 1.52, range 13–20 years). All participants were Caucasian.

### Measures

#### Depressive Experiences Questionnaire for Adolescents (DEQ-A)

The DEQ-A (60) is a 66-item self-report questionnaire, in which items are scored on a 7-point Likert scale, ranging from 1 (strongly disagree) to 7 (strongly agree). The original factor weighting coefficients were used in the present study (61). The DEQ-A scoring program yields three scales: dependency, self-criticism, and efficacy. In the present study, we considered only the DEQ self-criticism and dependency scales. In our sample, internal consistency was found to be moderate to good (α_dep_ = 0.61; α_sc_ = 0.82). The Italian version of the DEQ-A was developed using the back-translation method. This method achieves conceptual and cultural equivalence, as well as linguistic equivalence. First, a bilingual translator from the Department of Psychology translated the instructions and items of the original version into Italian. Next, the Italian version was back-translated into English by another bilingual translator from the Department of Humanistic Sciences. Finally, the original version was compared with the back-translated. The measure was reviewed by two Italian expert psychologists who had lived for at least 2 years in the United States. These experts contributed to the cultural adaptation of the questionnaire from American English into Italian. Where discrepancies occurred in the back-translations, the translators and the experts held discussions and worked cooperatively to make corrections to the Italian version. No items were eliminated or significantly adjusted during the translation process (62).

#### Attachment Style Questionnaire

The Italian version (63) of the Attachment Style Questionnaire [(ASQ); (64)] was used. It is a 40-item self-report scale containing five subscales that assess (a) adult secure attachment (*via* the confidence subscale), (b) insecure anxious attachment (*via* the Need for approval and the preoccupation with relationships subscales), and (c) insecure avoidant attachment (*via* the discomfort with closeness and relationships as secondary subscales). The factor structure has been reproduced among various community and psychiatric samples (51). All items are rated on a 6-point Likert-type response format ranging from 1 (totally disagree) to 6 (totally agree). In the current study, the 10-item discomfort with closeness, the 8-item confidence in self and others, and the 8-item relationships as secondary subscales were used as indicator variables for the attachment avoidance latent factor, whereas the 7-item Need for approval and the 7-item preoccupation with relationships subscales were used as indicator variables for the attachment anxiety latent factor. The Cronbach alpha coefficients for the five subscales ranged from 0.62 for Confidence in Self and others to 0.76 for Need for approval.

#### Suicidal Behaviors Questionnaire-Revised (SBQ-R)

Suicidal behaviors were assessed by SBQ-R (65). The SBQ-R is a 4-item measure of lifetime suicide ideation and attempts (“Have you ever thought about or attempted to kill yourself?”), frequency of suicide ideation in the last year (“How often have you thought about killing yourself in the past year?”), threat of suicidal behavior (“Have you ever told someone that you were going to commit suicide, or that you might do it?”), and likelihood of future suicidal behavior (“How likely is it that you will attempt suicide someday?”).

Respondents are asked to answer each question in terms of the frequency with which they engaged in the suicidal behavior, using a Likert-type scale. For example, respondents are asked to indicate their frequency of having suicidal ideations, ranging from 1 (never) to 5 (very often). Scores on the SBQ-R have been found to differentiate between suicidal and non-suicidal adults (65). In the present sample, internal reliability for the SBQ-R was 0.73. Higher scores on the SBQ-R are indicative of greater suicidal behaviors.

The Italian version of the SBQ-R was developed using the back-translation method following the process described above for the DEQ-A.

### Procedure

Students were asked to participate in a research study as volunteers. During class time and in groups of 25–30, students received a brief explanation about the purpose of the study. They subsequently completed the questionnaires. All students were given the possibility to call the Department of Psychology for subsequent information about the research. Participants gave written informed consent. In the case of students under the age of 18, their parents also gave written informed consent. Three administration sessions were required to obtain the sample.

The research protocol was approved, according to the Declaration of Helsinki and its revisions (66), by the Institutional Review Board of the University of Palermo.

### Statistical Analysis

We used structural equation modeling (SEM) (67) to test our hypotheses as these analyses can evaluate *a priori* models, suggest causal sequences, identify mediators, and elucidate direct and indirect paths. We examined the link between participants’ attachment style and their current suicidal behaviors, as well as the mediating role of self-criticism and dependency in this association. This allowed us to evaluate the association between attachment styles, self-criticism and dependency, and suicidal behaviors. SEM analyses were performed with the AMOS software [version 18.0; (68)] using maximum-likelihood estimation.

After verifying the univariate normality of the distributions using the Skewness and Kurtosis indices, the Kurtosis multivariate Mardia coefficient was used to test the multivariate normality between the variables (69). Then, we calculated the descriptive statistics for each variable, as well as bivariate correlations.

We examined the influence of all demographic variables. In particular, we explored the invariance across gender to determine whether gender might serve as a confounding variable related to the main analyses. A multiple-group analysis was conducted to check whether effects were equivalent across females and males (70).

Regarding SEM analyses, in addition to the overall χ^2^ test of exact fit, as suggested by Browne and Cudeck (71) and Hu and Bentler (72), the following fit indices were used to evaluate the proposed models: comparative fit index; values of 0.95 or greater are desirable, the standardized root-mean-square residual; values of 0.08 or less are desirable, and the root-mean-square error of approximation; values of 0.08 or less are considered to be reasonable.

Finally, since AMOS only provides bootstrap estimates, SEs and confidence bounds for total indirect effects [e.g., the sum of all specific indirect effects; (73–75)], mediation analyses were conducted using the PROCESS macro designed for SPSS (76) to test specific indirect effects. This macro uses bootstrapped sampling to estimate the indirect mediation effect. In this analysis, 1,000 bootstrapped samples were drawn and bias corrected 95% bootstrap confidence intervals (CIs) were reported. CI that do not include 0 indicate a significant indirect effect of the independent variable on the dependent variable through the mediators (76). Standardized betas are reported.

## Results

### Preliminary Analyses and Descriptive Statistics

We examined the influence of all demographic variables (age, education, school class, area of study, parental education and job, family income, and parental marital status) and found no significant relations with any study variables.

Gender analyses revealed significant mean level differences for all variables. Specifically, males scored higher in confidence (*p* < 0.001) and relationships as secondary (*p* < 0.05), whereas females scored higher in all the other variables (*p* < 0.01).

Then, we examined the invariance of the full model across genders to determine whether sex might moderate any of the paths in our model. Two models (a freely estimated model and a constrained model) were used to determine whether regression estimates varied significantly across genders. The freely estimated model was allowed to estimate regression paths and the structural covariances among factors without restriction, whereas, in the constrained model, factor loadings and the structural covariances among factors were constrained to be equal across the female and male groups. When the fits of the constrained and the unconstrained models differ significantly, this suggests at least some paths differ significantly between groups. This constrained model did not yield a significantly different fit than the unconstrained model, Δχ^2^ (10, *N* = 340, *p* > 0.05) = 8.51. This suggests that, in our study, the relationships between attachment styles, Blattian variables, and suicidal behaviors were not moderated by gender; nonetheless, since we had found that gender was related to some variables at a mean level, subsequent mediation analyses were conducted controlling for gender.

The expected correlations among all variables were statistically significant (*p* < 0.05), with the exception of the correlation between dependency and self-criticism (*r* = 0.03, *ns*), confidence (*r* = 0.02, *ns*), discomfort with closeness (*r* = −0.04, *ns*), and suicidal behaviors (*r* = −0.05, *ns*). Finally, the association between relationships as secondary and preoccupation with relationships was not significant (*r* = 0.01, *ns*).

Table 1 presents the descriptive statistics and correlations between the study variables.

**Table 1:** **Summary of intercorrelations, means, SDs, and alpha values for scores on the study variables**.

Variables	1	2	3	4	5	6	7	8
1. Dependency								
2. Self-criticism	0.03							
3. Confidence	0.03	−0.42^a^						
4. Discomfort with closeness	−0.04	0.41^a^	−0.34^a^					
5. Relationships as secondary	−0.23^a^	0.24^a^	−0.17^a^	0.25^a^				
6. Need for approval	0.30^a^	0.51^a^	−0.37^a^	0.39^a^	0.24^a^			
7. Preoccupation with relationships	0.36^a^	0.50^a^	−0.23^a^	0.29^a^	0.01	0.54^a^		
8. Suicidal behaviors	−0.05	0.34^a^	−0.30^a^	0.20^a^	0.14^a^	0.27^a^	0.16^a^	
M	−0.60	−0.18	30.62	37.95	16.53	22.32	30.38	7.14
SD	0.77	1.09	5.59	7.41	5.86	6.94	7.12	2.47
*α*	0.61	0.82	0.62	0.66	0.72	0.76	0.72	0.73

*^a^Correlation is significant at the 0.01 level (two-tailed)*.

### Blattian Variables As Mediators between Attachment Style and Suicidal Behaviors

**Figure 1:**
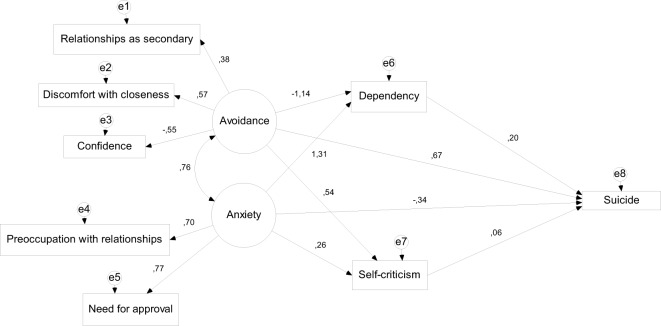
**The mediating role of dependency and self-criticism in the relation between attachment styles and suicidal behaviors**.

Despite a significant chi-square [χ^2^ (14) = 51.54, *p* < 0.001; χ^2^/df = 3.68], fit indices [RMSEA = 0.09 (90% CI = 0.064; 0.116), CFI = 0.94, SRMR = 0.05], the model showed an acceptable fit (Figure 1).

Greater attachment anxiety predicted greater dependency (β = 0.39, *p* < 0.001) and self-criticism (β = 0.67, *p* < 0.001). Furthermore, greater self-criticism (β = 0.22, *p* < 0.01) predicted increased while greater dependency (β = −0.14, *p* < 0.05) predicted fewer suicidal behaviors. The indirect effects of attachment anxiety on suicidal behaviors through both self-criticism [*point estimate* = 0.15 (95% CI: 0.06, 0.25)] and dependency [*p.e*. = −0.05 (95% CI: −0.11, −0.01)] were significant.

Attachment avoidance predicted lower levels of dependency (β = −0.17, *p* < 0.01) and greater levels of self-criticism (β = 0.71, *p* < 0.001). Self-criticism was also significantly associated with increased suicidal behaviors (β = 0.19, *p* < 0.05) although dependency was not (β = −0.05, *p* > 0.05).

The indirect effect of attachment avoidance on suicidal behaviors through self-criticism was significant [*p.e*. = 0.13 (95% CI: 0.04, 0.23)], although the same indirect path *via* dependency was not [*p.e*. = 0.01 (95% CI: −0.01, 0.03)].

## Discussion

The present study examined whether self-criticism and dependency mediate the relationship between attachment anxiety and avoidance and suicidal risk among adolescents. The current results support the expected mediation effects. Overall, both self-criticism and dependency were significant mediators in the relationship between attachment anxiety and suicidality, whereas only self-criticism mediated the relationship between attachment avoidance and suicidality.

This result, taken together with previous theory and evidence [e.g., Ref. (22, 36, 77–79)] indicates that a self-critical personality style, characterized by substantial sensitivity to criticism by others and to their own self-scrutiny and critical judgment (21), is a significant risk factor for engaging in suicidal ideation and behavior.

Specifically, results supported the prediction that self-criticism would mediate the link between both attachment anxiety and avoidance and suicidality, whereas, we found that dependency mediates the link between attachment anxiety and suicidality (52, 55). According to Cantanzaro and Wei (53), this finding can be explained by the following scenario: individuals with higher levels of attachment anxiety tend to develop a negative internal working model of the self (45, 80) and to automatically engage in self-criticism and harsh self-evaluation with the aim of reducing the likelihood of being criticized by others (81). A suicidal attempt could be explained as the outcome of the emotional breakdown, which is a consequence of the failure of this strategy, particularly when others show critical or harsh attitudes (20), from which the individual concludes they are fundamentally worthless and do not deserve to live.

Those with higher levels of attachment avoidance tend to develop a negative view of others and may develop beliefs surrounding the need to be highly competent or nearly flawless at life tasks in order to maintain self-reliance rather than risk further rejection (58). Suicide could be a reaction to interpersonal rejection and/or to failing to reach these excessively high standards.

Regarding the association between attachment anxiety, dependency, and suicidality, results indicate a significant mediational negative effect for dependency, indicating that dependent people seem to be less at risk for suicide. The major features of attachment anxiety are the desire for interpersonal closeness and the fear of interpersonal rejection or abandonment (56), as well as a negative internal working model of the self. Therefore, these individuals may develop a dependent tendency in order to ensure others’ availability and validation (52). Moreover, suicide would be the ultimate form of cutting close emotional ties, of which dependent individuals are quite afraid. This may explain why they use less lethal methods. That is, they do not want to die; rather, they want help from and to maintain close ties to others.

In line with hypotheses, regarding the association between attachment avoidance, dependency, and suicidality, results do not indicate a mediational effect for dependency. There are multiple possible explanations for this lack of an effect.

First, this finding is consistent with literature showing an unclear role of interpersonal vulnerabilities (i.e., dependency) in suicidality (40). Second, according to previous research [e.g., Ref. (52)], attachment avoidance and dependency show a negative association. Those with higher levels of attachment avoidance, in order to protect themselves against anticipated rejection, are likely to develop a tendency of not relying on others who are likely perceived as unavailable. Third, dependency is a multifaceted construct that is more weakly related to negative outcomes compared to self-criticism and which has been used to describe a wide array of personality traits by investigators from different fields of inquiry (21, 82, 83). As such, specific facets of dependency may be differentially related to suicidality relative to others.

This study has a number of limitations. First, the exclusive use of self-report measures may have inflated effects due to shared method variance. Further, such measures are susceptible to response bias, despite all being validated against more robust, contextual, interview-based approaches. This study is also limited by the cross-sectional design. Longitudinal studies are needed to investigate whether the clinical variables studied are associated over time as well as the direction of the relationship between them. Third, a relatively brief assessment of attachment styles was used which gives a continuous score of attachment insecurity rather than categorical styles. Finally, a community sample was used in the present study. Although this approach carries many advantages for research into developmental psychopathology (84), it limits our ability to make clinical inferences. Findings should be replicated in other samples with other measures of attachment style.

## Author Contributions

All authors participated in the concept and writing of this manuscript. All authors approved the final version of the manuscript.

## Conflict of Interest Statement

There are no potential conflicts of interest or any financial or personal relationships with other people or organizations that could inappropriately bias conduct and findings of this study.
